# Development and preliminary evaluation of a decision coach training module for nurses in Norway

**DOI:** 10.1186/s12912-024-02569-6

**Published:** 2025-02-10

**Authors:** Simone Kienlin, Kari Nytrøen, Jürgen Kasper, Dawn Stacey

**Affiliations:** 1 Department of Health and Caring Sciences, Faculty of Health Sciences, University of Tromsø, Postbox 6050, Langnes, Norway; 2 E-Health, Integrative Care and Innovation Center, University Hospital of North Norway, Postboks 100, Tromsø, 9038 Norway; 3 Department of Medicine and Healthcare, The South-Eastern Norway Regional Health Authority, Postbox 404, Hamar, N-2303 Norway; 4 Faculty of Medicine, University of Oslo, Blindern, Postbox 1072, Oslo, N-0316 Norway; 5 Department of Nursing and Health Promotion, Faculty of Health Sciences, OsloMet Metropolitan University, Pilestredet 46, Oslo, 0167 Norway; 6 School of Nursing, University of Ottawa, 451 Smyth Road, Ottawa, ON K1H 8M5 Canada; 7 Ottawa Hospital Research Institute, 200 Lees Avenue, Ottawa, ON K1S 5L5 Canada

**Keywords:** Shared decision-making, Decision coaching, Curriculum development, Decision support techniques, Nursing education, Communication skills

## Abstract

**Background:**

Shared decision-making (SDM) is a collaborative patient-centred process for arriving at informed healthcare decisions. Decision coaching can help support SDM when combined with patient decision aids. As part of a meta-curriculum “Ready for SDM” for training different healthcare professionals in SDM, we developed and pilot-tested a new module designed to train nurses as decision coaches. The study assessed nurses' perceptions of a decision coach training module, focusing on its feasibility, acceptability and its role in developing decision coaching capabilities.

**Methods:**

We used a two-phase approach guided by the Knowledge-to-Action Framework. In the first phase, we developed a decision coach training module. The second phase involved preliminary testing, using a descriptive design with qualitative and quantitative methods. We recruited a convenience sample of participants from two hospitals. Participants completed questionnaires at the end of Part A (classroom training). The assessment was informed by Kirkpatrick’s first three levels of educational outcomes: reaction (acceptability), learning (self-reported attitudes, intentions and confidence) and behaviour (practical application of decision coaching). A post-hoc inquiry investigated low participation in Part B of the coach training. Qualitative data underwent content analysis and quantitative data were analysed using descriptive statistics.

**Results:**

The development resulted in a decision coach training comprising a Part A (6 h) on SDM and decision coaching fundamentals and a Part B (1 h) which involved practical application of decision coaching in the participants’ own practice (audio recorded) with self-appraisal and individualised feedback. In preliminary testing with 19 nurses from seven clinical departments, 90% of participants rated Part A as acceptable and relevant to practice. Only one nurse completed Part B due to reluctance to audio record coaching sessions. The most reported perceived barrier was time constraints. Key perceived facilitators identified were interprofessional collaboration, management support and additional practical training.

**Conclusion:**

Decision coach training was feasible to deliver in the classroom. Participants reported Part A as acceptable and relevant to their practice. The second part, including an audio recording component, proved unfeasible. Further research should explore alternative methods for skill assessment and feedback in clinical practice. The results from this study will inform further refinement of the Norwegian Ready for SDM meta-curriculum and implementation strategies, particularly regarding the practical training components.

**Trial registration:**

Retrospectively registered (14.02.2023) at ISRCTN (ISRCTN44143097).

**Supplementary Information:**

The online version contains supplementary material available at 10.1186/s12912-024-02569-6.

## Background

The Norwegian Patients’ Rights Act emphasises patients’ right to be involved in healthcare decisions, aligning with the national motto “The patients’ healthcare services” [1–3]. Shared decision-making (SDM) is a collaborative, patient-centred process for arriving at informed values-based decisions in the context of treating, screening, or preventing health conditions. This approach is used in preference-sensitive decisions, where there is more than one treatment option and/or the best choice depends on how the patient values the potential benefits and harms [4]. SDM has been shown to improve patient outcomes and experiences [5, 6] and several interventions have been developed to facilitate the use of SDM [7–10]. Patient decision aids [6] and decision coaching [11] are examples of interventions used to facilitate SDM.

PDAs, such as videos, printed materials and, web-based formats, are tools developed to support patients and healthcare providers (HCPs) make informed decisions. They do this by clearly stating the decision to be made, providing evidence-based information on the options and outcomes and clarifying personal values [6]. PDAs are designed to complement, not replace, counselling from HCPs. Research suggests that combining shared decision making training with patient-targeted interventions like PDAs can increase HCPs’ use of SDM [7]. PDAs are being developed and published on the Norwegian national platform Helsenorge.no, where patients can also find their personal, registered health information and several digital services for following their health [12]. However, PDAs and SDM have not yet been routinely implemented in clinical practice [13–15]. Hence, there is a need to develop additional interventions to support HCPs to involve patients in health decisions.

Decision coaching is an intervention provided by trained HCPs supporting patients in the process of making informed decisions through non-directive counselling and encouraging the patient to develop or enact decision-making skills such as: understanding risk information, thinking about the options, expressing preferences and concerns, discussing a decision with healthcare professionals and implementing a chosen option [16, 17].

Nurses, social workers, or other allied health professionals can provide decision coaching, which, when combined with evidence-based information, may improve patients’ knowledge [11]. Some studies have shown that nurses in certain medical specialities, acting as decision coaches, have successfully enhanced patient involvement in making health decisions [18, 19]. Hence, nurses may play decisive roles in supporting patient participation in decisions regarding their own health, but nurses need the knowledge and skills to do so [16]. For example, nurses require skills in values clarification and knowledge about providing evidence-based health information to effectively offer decision support. The most frequently reported barrier to implementing SDM in hospital settings was “limited skills”, including lack of training in SDM skills [20].

In the context of the Norwegian health system’s efforts to build an overarching strategy to establish a culture of SDM, a meta-curriculum entitled “Ready for SDM” (*Klar for samvalg* in Norwegian) has been developed [21–25]. The Ready for SDM comprises several SDM training modules, using both classroom and online formats, as well as guidance for tailoring the modules to the different contexts and needs of HCPs. Several modules have been proven to be feasible and effective for changing SDM competencies [21–26]. However, a critical gap remains: none of these modules specifically address the unique role of nurses as decision coaches.

In the current study, we aimed to develop and conduct preliminary testing of a decision coach training module. The study addressed three primary research questions: 1) Is the decision coach training module feasible to deliver and acceptable to nurses? 2) Does the training enhance participants' self-reported knowledge and skills needed for decision coaching? 3) What are the perceived barriers and facilitators for implementing decision coaching and decision aids in clinical practice?

## Methods

We used a two-phase approach, guided by the Knowledge-to-Action Framework [27]: 1) Developing of the decision coach training and 2) conducting preliminary testing with a descriptive design using both qualitative and quantitative methods. The KTA Framework [27] integrates two major concepts, knowledge creation and the action cycle. Knowledge creation involves identifying knowledge relevant to the practice problem targeted for change and includes creating knowledge tools, such as training, to support implementation in the action cycle. The action cycle comprises seven steps that facilitate a guided approach to practice change: identifying the evidence-practice gap (e.g., inadequate knowledge/skills in decision coaching), adapting knowledge to context (e.g., adapting training for target audiences), identifying barriers and enablers, selecting and tailoring interventions to overcome identified barriers, monitoring use, evaluating and sustaining the change. Using the KTA, we adapted training components from previously developed SDM training and developed new components.

Behaviour change techniques (BCTs) were used in the intervention as a method to increase use of decision coaching. The use of BCTs contributes to transparency and improves the replicability of SDM interventions [28, 29].

To enhance accurate and complete reporting of this study, the STROBE guideline [30] was followed where applicable (Additional file 1).

### Phase 1: Development of the nurse training curriculum

#### Rationale for the training and learning objectives

We developed a decision coach training module to be added to the Ready for SDM meta-curriculum. The purpose of the decision coach training was to build knowledge and skills needed to provide decision coaching to patients. There were 10 specific objectives for Part A in the classroom instruction and five objectives for Part B on the self-appraisal of decision coaching (see Table 2). To accomplish these objectives, we selected relevant modules from the meta-curriculum (Basic SDM course and SDM skills in situ), components from other evaluated coaching trainings and created a new module specific to decision coaching.

Rationale for the training also considered the nurses’ motivation to engage in becoming a decision coach. Motivation might be strong due to nurses’ closeness to the patient, their particular focus on trust and relationship building and their central roles as providers of treatment and counselling [31]. On the other hand, nurses might be reluctant to become committed to tasks related to decision-making, which they might feel belong to the traditional responsibilities of the physicians. The training was designed to target nurses from quite heterogeneous medical contexts. To equip trainees with skills for specific contexts while training them in context-heterogeneous groups puts particular emphasis on the need to facilitate the transfer from generic knowledge and skills to the individual decision settings and medical contexts. The development was focused on nurses working in a medical domain with existing PDAs to make sure that the nurses would have access to evidence-based information for a specific decision for use with patients, which is known to improve outcomes of decision coaching [11].

#### Development of the decision coach training

In line with the purpose of the “knowledge creation funnel” of the KTA framework, the draft for the decision coaching training was adapted from our group's previous studies evaluating SDM training [18, 21, 22, 26, 32–34]. The module's design was shaped by Knowles' Adult Learning Theory [35], which highlights four crucial planning steps for adult learning: 1) determining learning needs, 2) creating appropriate learning strategies and resources, 3) implementing these strategies and 4) evaluating outcomes. This framework aligns seamlessly with our development process, where learning needs were initially assessed through stakeholder feedback, during the piloting of components and a brief literature review of learning objectives for nurses in SDM [36]. Resources were then tailored to meet these needs and finally, outcomes were evaluated using Kirkpatrick's model.

The curriculum is designed for a structured progression from foundational knowledge to advanced competencies in SDM and decision coaching. Initially, participants grasp basic SDM principles and coaching concepts. As they progress, they develop practical techniques and communication skills through role-play and simulations in Part A of the module. Part B advances to independent application and self-appraisal in clinical settings. Each stage builds on the previous, enhancing participants' abilities to apply learned skills in real-world scenarios. This progression aligns with Bloom's Taxonomy, moving systematically from basic knowledge and comprehension to advanced application [37]. Table 2 clearly outlines this progression, aligning learning objectives with corresponding learning/teaching methods and materials to ensure a coherent educational progression.

**Fig. 1:**
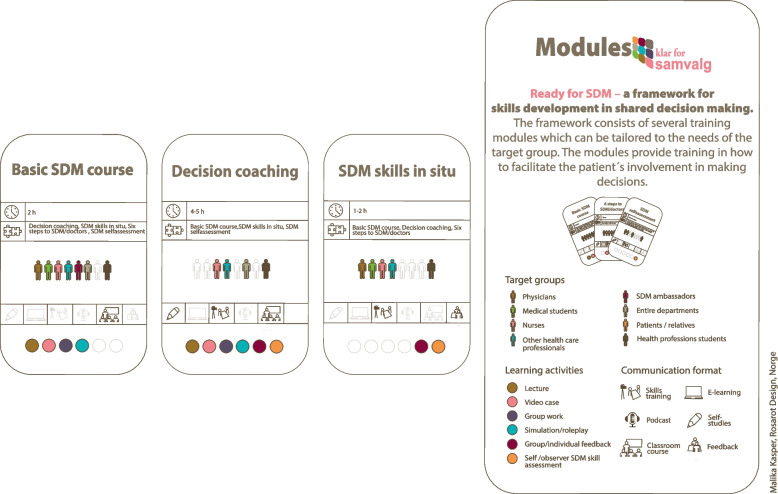
“Decision coaching for nurses” module: duration, groups targeted, learning activities and communication formats. The figure presents a visual overview of the training modules’ time span, compatible modules, target groups and learning activities and communication format. Illustration: Malika Kasper, for "Ready for SDM". South-Eastern Norway

Two established modules from the meta-curriculum were incorporated in the new decision coaching training module: 1) a generic “basic SDM course” (Fig. 1), previously evaluated for this target group and 2) the proven efficacious observation- and feedback-based “SDM skills training in situ” from the doktormitSDM module (new name: SDM skills in situ). In addition, we created a new module specific to decision coaching. The single components in the “decision coaching” part, regarding the nurse’s role in interprofessional SDM, were developed or adjusted, inspired by a Canadian decision coach training approach [32, 38] in the associated Framework for Decision Coach-Mediated Shared Decision Making (based on the Ottawa Decision Support Framework) [17].

Single components of the decision coach training, such as role-play exercises and a moderated discussion of nurse-led decisions relevant for SDM, were piloted within several training sessions for nurses guided by the KTA. Although the action cycle from the KTA framework has a stepwise flow, the phases were revisited several times before moving on to later phases. For example, after piloting the role-play exercises, we returned to the “knowledge creation funnel” to develop new methods for overcoming the barriers that were encountered. This resulted in the development of standardised patient cases (Table 3). Exercises to facilitate the training of practical skills were adjusted from the Canadian approach to fit the Norwegian context. Field observations were taken by the instructors to assess the appropriateness and comprehensibility of the components.

Learning resources and BCTs [29] used in the Ready for SDM meta-curriculum [22, 39] were adapted for nurses providing decision coaching. Throughout the entire training, learning activities were used with reference to evidence-based BCTs. BCTs are considered the “active ingredients” in the implementation of an intervention and are defined as the observable, replicable components of behaviour-change interventions that can be used individually or in combination with other BCTs [29, 40]. For example, the BCT “action planning guidance” in the meta-curriculum was operationalised using pocket reminder cards (see Table 1) [24].

*We applied to the* Norwegian Nursing Association (NSF) for six hours of *Continuing Nursing Education Credits. A* certificate of completion of the module (parts A and B) was approved by the Norwegian Nursing Association (NSF) for six hours of *Continuing Nursing Education Credits*.

### Results of phase I

The intervention, structured according to the Template for Intervention Description and Replication (TIDieR) checklist [41] (see Additional file 2), featured a specifically designed decision coaching module for nurses in Norwegian hospitals. The module was scheduled as a one-day event, comprising six hours of classroom training, followed by a 30-min practical exercise in decision support in their own clinical setting with a 30-min follow-up feedback session (Table 1).

**Table 1:** Behaviour change techniques (BCTs) used in the decision coaching module

BCTs used in the decision coaching module	Operationalisation of the BCTs in the decision coaching module
Problem solving (1.1) Tailoring (Agbadjé 2020)^a^	Exercises and group discussions were used to draft solutions for interprofessional role distribution in typical domain-specific decision scenarios
Action planning guidance (1.4)	Detailed planning of when to conduct decision coaching while using a decision aid The 6 steps to SDM on “pocket reminder cards” (handout)
Feedback on behaviour (2.2)	Feedback on the audio recordings and role-play provided
Instruction on how to perform the behaviour (4.1) Demonstration of the behaviour (6.1)	Suggested structure for consultations involving patients provided using: the 6 steps to SDM “pocket reminder cards” Example videos, role-play by the instructor, a presentation on nurse-led DC
Information about health consequences (5.1)	Information was provided about the effects of SDM on patient-relevant outcomes
Information about social and environmental consequences (5.3)	The training referred to national and local policies and ethical guidelines for implementing SDM
Information about others’ approval (6.3)	Information provided about the demand for nurse-led SDM from patients and government focus on its importance
Behavioural practice (8.1)	Role-play with pre-prepared cases (Part A) and providing decision coaching to a patient with decisional needs (Part B)
Generalisation of a target behaviour (8.6)	Encouragement to use the decision coaching approach after the training and in all SDM-relevant decisions
Use of a credible source (9.1)	Evidence on patients’ preferences to take control of their health choices and of HCPs’ flawed assumptions about SDM was provided Evidence on patients’ ability to process evidence-based information provided Evidence challenging the claim that SDM is too time-consuming provided
Restructuring the social environment (12.2)	Advice on restructuring the information flow and interprofessional collaboration to promote patient involvement
Adding objects to the environment (12.5)	Trainees introduced to PDAs and the OPDG Tools for risk communication Pocket reminder cards

Numbers in brackets refer to the BCT taxonomy by Michie (2013). Otherwise, another reference is provided (^a^)

### Part A: Decision coaching skills (6h)

In the first two hours (basic SDM course, Fig. 1), participants were introduced to the background and rationale for SDM, the situations where SDM is relevant, risk communication techniques and measures for supporting SDM such as PDAs (Table 2). Six steps structuring an SDM process were presented by providing a detailed description of several levels of performance for each quality indicator [42] and by showing video cases from real encounters and an exercise with standardised case vignettes (Table 2).

**Table 2:** Learning objectives and content of the decision coach module for nurses

**Part A:** 6-h classroom training				
**Learning objectives**	**Bloom's Level**	**Content**	**Learning activities**	**Learning materials**
• Know background and rationale for SDM and decision coaching (DC) • Understand informed choice • Identify the situations in which SDM/DC is relevant	• Remember • Understand	• Background and rationale for SDM/DC • Documented effects of SDM/DC • *Informed choice* • *Criteria for identifying decisions relevant for SDM* and assessing decisional needs • *Models of patient participation and SDM* • *Criteria for appraising the quality of risk communication*	• Lectures • Group work • Think-pair-share (TPS)	• *SDM animation video* • *“Identify relevant decision for decision coachingexercise”* • Patient narratives
• Explain and justify the structure of an SDM process its quality criteria	• Analyse	• *Six steps that structure an SDM -process* • *SDM quality criteria* • Roleplay provided by the instructors	• Lecture • Video of an SDM case • Reflection exercises	• Videos of clinical encounters • Pocket reminder card (showing SDM steps) • Standardised case vignettes
• Know effective measures for supporting patients in making decisions	• Remember	Methods to support SDM: • *Presentation of numbers* • *Teach back* • *PDAs* • The Ottawa Personal Decision Guide (OPDG) • *Six steps structuring an SDM process*	• Lecture • Practical examples	• Diagrams • PDAs on the national platform • OPDG • Pocket reminder card (showing SDM steps)
• Describe the nurse’s role in SDM • Identify decisional needs	• Understand • Apply	• The nurse's role in interprofessional-SDM (IP-SDM) • Draft of solutions for interprofessional role distribution in typical domain -specific decision scenarios • Exploration of decision needs such as knowledge gaps, unclear values, lacking support or uncertainty	• Lecture • Video of an SDM case • Group discussion • TPS	• Videos of cases from clinical encounters • Needs assessment exercise
• Navigate easily through the 6 steps of an SDM process • Internalise skills for coaching patients through the 6 SDM steps for medical problems in their specialty	• Apply	• *Six steps structuring an SDM process* • Conduct simulated decision coaching • Adaptation of SDM principles to different SDM relevant decisions	• Case-based decision coaching simulation/roleplay • Group/individual feedback	• Pre-prepared DC cases (see Table 1) • Pocket reminder card (showing SDM steps) • PDAs on the national platform • OPDG
• Know the answers to frequently asked questions/barriers about SDM	• Remember	• *Typical barriers to the implementation of SDM—equipping participants with reasonable counterarguments*	• Facilitated discussion using “barrier cards”* • Group discussion	• Barrier cards*
**Part B: Decision coaching in practice: support a patient with decisional needs and receive individualised feedback**				
**Learning objectives**	**Bloom's Level**	**Content**	**Learning activities**	**Tools/models**
• Internalisation of the nurse’s role in SDM	• Apply	• Provide decision support to a patient with decisional needs while audio-taping the encounter	• Self / observer SDM skills self/observer assessment • Individual feedback	• MAPPIN´SDM
• Acquire decision coaching skills comprising the assessment of decisional needs and providing decision support, using PDAs or OPDG • Explain and justify the structure of an SDM process and its quality criteria • Navigate easily through a decision-making process • Learn to self-evaluate decision support skills	• Apply/create • Analyse • Evaluate	• Provide decision support to a patient with decisional needs • Transfer SDM principles to different SDM relevant decisions	• SDM skills self/observer assessment using audio-recording • Individual feedback	• Audio-recorder • MAPPIN´SDM • PDAs on the national platform • OPDG

Italic text refers to content within the “basic module” and the “SDM in situ” module previously developed and reported elsewhere

*SDM* Shared decision making, *DC* Decision coaching, *MAPPIN´SDM* a set of measurement scales assessing communication quality in SDM [13, 35], *TPS* (*Think*-*pair*-*share)*, a cooperative learning activity that involves individual thinking, collaboration, and then presentation, *Teack Back method*: is a way of checking understanding by asking patients to state in their own words what they need to know or do about their health, *PDA* Patient decision aid, *OPDG* Ottawa Personal Decision Guide [36], *Barrier cards* Laminated cards containing answers to various barriers, using a generic technique to meet the barriers (acknowledging, rephrasing, information, argument and cognitive restructuring)

Bloom's Taxonomy Levels Used in Training [37]

The subsequent four hours focused on the Decision Coaching module (Fig. 1), where instructors demonstrated decision coaching behaviours through role-play, showcasing both suboptimal and optimal examples. The role of nurses within the interprofessional SDM process was emphasised, discussing how to operationalise behaviour change techniques such as "Instruction on how to perform the behaviour" and "Demonstration of the behaviour." Participants practised simulated decision coaching in pairs, assessing patients' decisional needs using the Norwegian version of the Ottawa Decision Support Framework [43], supported by pocket reminder cards outlining the SDM steps tailored to the decision coach role [19, 22].

For the next interactive learning exercise, participants used the Ottawa Personal Decision Guide (OPDG) [44] or PDAs on the national Norwegian platform [12]. Then they engaged in roleplay exercises using standardised patient cases (Table 3). Participants practiced decision coaching tailored to these specific simulated patient needs, using the OPDG or PDA relevant to their clinical specialty and received feedback from the instructor.

**Table 3:** Examples of patient cases for role-play

Underlying issue	Patient version of the case	Decision Coach version of the case
The “insecure” patient -Feels uncertain due to lack of information/ understanding	• You find there is a lot of information that is difficult to understand • You do not feel ready to take part in the decision • You have not completed the whole PDA because you felt overwhelmed	Description of the patient: The patient finds a lot of the information is difficult to understand. They do not feel ready to make the decision Tips for coaching: • Check the patient's health literacy level and understanding. Feel free to use the teach-back method^a^ • Explain the effects of treatment using plain language. Use repetition and formulate the message in different ways
The “respect-for-authority” patient -Thinks the nurse knows best	• You read the contents of the PDA thoroughly, but think that providing this information/PDA is just a nice offer • You believe that nurses should make recommendations and make the decision for you	The patient reads the contents of the PDA thoroughly but thinks that providing this information is just a nice offer. The patient has a high respect for your knowledge and professionalism and wants you to decide for them Tips for coaching: • Spend time on the first part of the PDA that emphasises steps 1 and 2 (defining the problem and key SDM message) • Focus on the patients’ values for outcomes of options and coach them in sharing that with the doctor
The “already-made-up-their-mind” patient	• You have a neighbour who, a while ago, faced the same treatment decision. She recovered, so you have decided to choose the same treatment • You find information in the PDA about your neighbour’s treatment. You look for confirmation that it’s a good choice and don’t bother with the other options	The patient has decided to choose the same treatment as her neighbour, who has recovered The patient selectively reads and finds only the information appropriate to her preferred treatment. She has not really understood the situation/information Tips for coaching • Check the patient’s understanding, especially of the parts in the PDA you suspect the patient has not read carefully • You need to guide the patient through a complete review of the PDA

^a^Teach-back method: is a way of checking understanding by asking patients to state in their own words what they need to know or do about their health

#### Part B: Decision coaching practice and personal evaluation (1h)

The second part of the training focused on practical application and evaluation. Three primary goals were for the nurses: to apply their knowledge and skills by providing decision coaching to an actual patient with decisional needs in their clinical practice, to analyse their own decision coaching performance and to receive individualised feedback on their recorded coaching session.

To facilitate this practical component, participants received an audio recorder, memory stick and patient informed consent form. They were instructed to record their decision coaching session and analyse it using the MAPPIN'SDM (Multifocal Approach to Sharing in SDM) HCP scale (see Additional file 3) [42, 45].

A follow-up meeting with the course instructor (SK) was arranged to occur either at the clinical department or via a virtual call. For those who submitted recordings, a 15-min interactive feedback session was provided, tailored to each individual nurse based on MAPPIN'SDM analysis of their audio taped decision coaching [26]. The MAPPIN'SDM inventory, which demonstrates good sensibility, reliability and validity [46], was used by SK to assess the extent of patient involvement in decision-making in the recorded coaching sessions. Rather than providing quantitative feedback, the instructor identified and discussed specific areas where further skill development would be most beneficial.

#### Phase II: Preliminary testing of the decision-coaching training module

## Method

### Study design

The study employed a descriptive design using both qualitative and quantitative methods. The evaluation framework was based on Kirkpatrick's four-level model for assessing educational outcomes, which includes: Level 1 (Reaction), Level 2 (Learning), Level 3 (Behaviour) and Level 4 (Results). This study focused on the first three levels, as measuring results (Level 4) was beyond the scope of this preliminary evaluation.

To address our three primary research questions, we aligned each question with the corresponding Kirkpatrick level: Level 1 (Reaction) assessed the feasibility and acceptability; Level 2 (Learning) assessed the development of perceived knowledge and skills; and Level 3 (Behaviour) examined the barriers and facilitators to implementation.

Nine months post-intervention a post-hoc inquiry to assess the barriers to completing audio recording was conducted.

#### Setting and participants

The study was conducted at two study sites (hospitals) within the Northern and the South-Eastern Norway Regional Health Authorities. The organisers were informed that the target group for the decision coach training was nurses working in departments with SDM relevant decisions (i.e. PDAs available such as breast cancer surgery or PDAs in development). The participants needed to be able to understand and communicate in Norwegian.

### Evaluation of the intervention

#### Study procedures

Recruitment was pursued through SDM ambassadors/facilitators who had previously undertaken the “Ready for SDM – train-the-trainer course”. They were informed about the accompanying evaluation of the training and they organised practicalities regarding the course. The training programme was designed for a maximum of ten nurses per course and was approved as regular working hours for the nurses.

Data collection was conducted at two time-points:

- T0: Post-classroom training (immediately after Part A): Collection of informed consent followed by self-administered online questionnaire.
- 4–6 weeks post-training: Reminders were sent at both four and six weeks after the training intervention to ensure participants recorded their practical application of decision coaching.
- T1: 9 months post-intervention: Nine months after the intervention, a post-hoc inquiry was conducted to identify barriers to completing audio recordings of practical applications of decision coaching. The course originator (SK) executed telephone interviews with two attempts per participant. For those who had not delivered an audio recording and were not reached after the second telephone call, an online survey was distributed by email. Additionally, a one-week follow-up reminder to complete the survey was sent to ensure comprehensive data collection.

The training module was documented in a training protocol to ensure standardised delivery in both locations. All training was delivered by SK, a registered nurse with a master’s degree in Health and Empowerment and a PhD student. She was trained in the using the Ottawa Decision Support Framework at the University of Ottawa in a graduate course and is a certified MAPPIN´SDM observer.

#### Outcome measures

Participants reported their demographics, including gender, age, clinical departments and years of clinical practise in a short questionnaire (Table 4). Variables measured to evaluate the module were based on three of Kirkpatrick's four levels [47], incorporating both quantitative and qualitative measures. The primary assessment tool was a self-administered online questionnaire comprising 13 items, adapted from previously validated evaluations used in other Ready for SDM curriculum modules (see Additional file 4). This was supplemented by the MAPPIN'SDM observer instrument, which measures the extent of patient involvement in decision-making. The items, provided at Kirkpatrick's Level one (Reaction), assessed relevance and satisfaction using four 4-point Likert-scale items (1 = strongly disagree, 4 = strongly agree) and one question had the response options "yes," "no" and "unsure". Additionally, indicators of applicability and suggestions for improvements were collected from the participants using open-ended questions and field notes were taken by the co-host.

**Table 4:** Characteristics of participants

		*N* = 19 (%)
**Sex**	Female	18 (95)
	Male	1
**Age**	< 30	1
	30–50 years	11 (58)
	> 50 years	7 (37)
**Years of clinical practice**	Fewer than 6 years More than 6 years	4 15
**Clinical departments**	Cancer in/outpatient clinic (*UNN)*	3 (15.5)
	Gastroenterological surgical department (UNN)	1 (4.2)
	Outpatient clinic for women's health and surgery (UNN)	1 (4.2)
	Dialysis department (UNN)	1 (4.2)
	Dialysis inpatient/outpatient clinic (Ahus)	7 (37)
	Breast and endocrine surgical outpatient clinic (UNN)	1 (4.2)
	Medical Outpatient Clinic, Centre for Obesity (UNN)	2 (10.5)
	Urology departments (in/outpatient clinic) (Ahus)	4 (21)

*UNN* University Hospital of North Norway, *Ahus* Akershus University Hospital

At Level two (learning), we assessed perceived understanding, attitude, self-rated SDM skills, self-confidence about providing decision coaching using five items (4-point Likert scale, 1 = strongly disagree, 4 = strongly agree) and one item regarding the commitment to providing decision coaching in clinical practice (5-point Likert response scale, 1 = Very likely, 5 = Very unlikely).

At Level three (behaviour/practical application of decision coaching), we assessed whether 1) the participants completed Part B and 2) the nurses’ perceived barriers and facilitators for implementing decision coaching in their context and their intention to implement it along with PDAs (two open-ended questions at the end of Part A).

### Data analysis

Descriptive statistics were calculated using SPSS version 26.0 (IBM Corporation, USA). Data from the post-intervention paper questionnaire were calculated using frequencies and reported as percentages of the answer categories (relevance, satisfaction, knowledge, attitude, confidence, commitment, age, duration of clinical practice). The qualitative data from free-text items were entered into NVivo version 11 (QSR International, Melbourne, Australia). NVivo was employed to maintain continuous access to the complete text linked with each identified code or category during the analysis process.

Analysis of free responses and field notes followed the principles of inductive qualitative content analysis as outlined by Hsieh and Shannon [48]. Two coders conducted the analysis using a step-by-step approach: 1) reading all data to achieve immersion, 2) performing open coding by highlighting keywords and phrases and 3) grouping similar codes into descriptive categories to provide a descriptive overview. Illustrative quotations were selected to exemplify the categories. Qualitative quotations originally in Norwegian were translated into English by the authors.

## Results

Our research questions addressed three key areas: feasibility and acceptability of the training, perceived knowledge/skills development and perceived barriers/facilitators to implementation. Analysis of quantitative and qualitative data revealed both the outcomes of the training and insights into its implementation. Qualitative analysis of free-text responses and field notes identified three main categories (training reactions, implementation barriers and facilitators).

### Participant characteristics

Nineteen nurses participated. Typical participants were female, aged 30 to 50 years, with over six years of experience working in a range of practice environments including cancer clinics, dialysis units, surgical departments and outpatient services (see Table 4 for complete details).

### Feasibility of training delivery

The training was successfully delivered in two hospital settings: the University Hospital of North Norway (UNN) in Tromsø (Northern Regional Health Authority) in December 2019 and at the Akershus University Hospital (South-Eastern Norway Regional Health Authority) in January 2020.

Part A (classroom component) was delivered as planned, with all 19 participants completing the full-day training. The training was conducted in hospital meeting rooms with participants engaging in both individual and group activities.

Part B (practical component) had limited completion, with only one participant conducting and recording a decision coaching session. This session addressed decision-making and lasted 22 min. Based on MAPPIN'SDM analysis, the participant received feedback on three areas for skill development: 1) enhancing problem definition by explaining treatment rationale and goals, 2) incorporating key SDM messaging about there being no single "right" choice and 3) when explaining the options, specifying the likelihood of benefits and harms to occur in an understandable way.

### Acceptability to participants

Field observations revealed that the nurses were engaged and in active dialogue, both in pairs and in plenary sessions. They verbally discussed the relevance of teaching to their practice and shared their own patient cases. They also discussed their challenges and barriers to providing decision coaching and applying PDAs in their own practices. Participants expressed that they liked the session and that it helped them envision how to apply the skills in practise.

Qualitative feedback supported these observations. Participants highlighted the applicability of “*learning how to use the PDAs*”, “*learning about the six-step structure of SDM*” and in general learning about decision coaching. Nine participants made suggestions for improving the training, “*including more time to review the PDAs before the role-play exercises”*, “*sending more information by email in advance of the training*”, “*having more participants from the same hospital unit”* and “*a larger room for the training due to noise during role play”*. Immediately after the course, 17 of 19 participants (90%) reported that what they learnt during the course was highly relevant to their job. Of 19 participants, 17 (90%) would recommend this course to nursing colleagues (with two participants being usure) as an indicator of being satisfied.

### Knowledge and skill development

Analysis of post-intervention questionnaires demonstrated that all participants (*n* = 19) perceived the concept of SDM as comprehensible, while 16 (84%) fully understood the concept of decision coaching, with three participants reporting partial understanding. Regarding attitudes towards patient involvement in healthcare decisions, 16 (84%) expressed positive attitudes, while three participants partially agreed.

Self-reported confidence levels varied across domains. Of 19 participants, 14 (74%) felt confident they could provide decision coaching within the next six months, while five participants reported uncertainty. In terms of skill assessment, 13 (68%) self-reported good decision coaching skills, while six (32%) reported limited coaching skills. Notably, confidence in PDA utilisation was lower, with only 3 (16%) reporting full confidence, 10 (53%) partial confidence and 6 (31%) expressing lack of confidence.

Regarding implementation readiness 12 of the 19 participants (63%) said that it was very likely or likely that they were able to provide decision coaching in their department, while six (32%) were unsure and one (5%) indicated unlikely.

### Implementation barriers and facilitators

Sixteen nurses provided 31 statements implementation barriers and facilitators in their clinical departments. Primary barriers included insufficient time allocation, motivational factors and confidence levels. One participant expressed skepticism about hospital-based PDA provision, stating: *"I think the patients can read the PDA at home, that's best for these patients. Many have decided before they come that they know what they want".* Another participant emphasised “*one has to have a suitable patient group and to feel that this PDA is the right tool to use for your patients".*

The post-hoc inquiry investigating low participation in Part B revealed multiple barriers. Of the 18 non-participating nurses, nine were successfully contacted. Five reported concrete plans for decision support implementation and audio recording, while four cited barriers including limited patient contact or perceived patient unsuitability. Several participants (*N* = 9) noted their course attendance was management-directed rather than self-selected, potentially affecting motivation: “*I did not choose the course myself but was recommended to attend it by my leader; this may have affected my motivation for performing the audio recordings*”. One participant articulated concerns about audio recording with vulnerable populations: "It is a vulnerable group, a lot of stigma, so I did not want to meet them with an audio recorder”.

Key facilitators identified through qualitative analysis included interprofessional collaboration, management support and additional practical training opportunities. Notably, the single participant who completed the audio recording, while reporting initial stress, continued utilising the pocket reminder cards in subsequent coaching sessions stating, “*It felt unnatural. I was stressed by having to make the recording, but I was very happy when I completed it and I have done several coaching sessions using the ‘pocket reminder cards’ since then*.”

## Discussion and conclusion

### Discussion

#### Principal findings

Guided by the KTA framework, this study demonstrated that it was possible to develop a module on decision coach training for nurses that could be used alongside the "SDM basic module" and the "SDM in situ" modules. Our findings revealed three key messages. First, the classroom component proved both feasible and acceptable, with nurses participating actively and 90% rating it as highly relevant to their practice. Second, while participants expressed high confidence and positive attitudes immediately after training, the module could not yet demonstrate behavior change in clinical practice. Third and most notably, we uncovered a significant barrier to skill development: nurses' strong reluctance to audio record their coaching sessions, with only one of 19 participants completing this crucial practical component.

The study has several possible limitations. Firstly, it is limited by the small sample; however,

the objective to assess the reaction, learning and mode of delivery of different components was reached. Secondly, while the study relied on non-validated questionnaires for evaluating training outcomes, this approach was chosen due to the need for tailored assessment measures that aligned with the specific learning objectives of the decision coaching training, a common necessity in educational intervention research [49]. Thirdly, even though the eligibility criteria were nurses working in a medical domain with existing PDAs, not all nurses had access to a PDA. For those not having access to a PDA, it may have created a feeling of not having the available support to implement decision coaching. The main limitation of the study was the overall lack of participation in Part B and thus, the missed opportunity to evaluate participants' decision coaching sessions in their clinical practice using audio recordings. On further inquiry, the nurses revealed other barriers that had prevented them from completing the module.

From a logical point of view, it would be reasonable to distinguish two main reasons why the nurses did not pursue the second, practical part of the training. First, barriers to implementing decision coaching in clinical practice and second, barriers specific to completing the training programme, particularly obtaining individualised feedback through audio recordings.

Regarding implementation barriers, although the decision coaching module was designed to overcome known barriers from the literature, there continue to be obstacles to nurses using it in practice. Research has identified common barriers that can impede decision coaching implementation at three levels: the patient level [50], the organisational level [16, 51] and the healthcare professional level [16, 52]. For example, not feeling one has the necessary information about all the alternatives, lacking communication skills, or holding strong opinions about what is best for an individual patient are potential barriers addressing nurses.

The "Ready for SDM" training modules address many of these barriers by giving them a voice in the classroom and by equipping participants with reasonable counterarguments. However, some of them cannot easily be remedied. Implementation of SDM is still constrained by information on benefits and harms not being accessible enough in a form that facilitates informed decisions, such as patient information following criteria for evidence-based health information or PDAs.

In Norway the development of decision aids is supported by the government [53] and several PDAs have been published on the central health platform “helsenorge.no.” However, the implementation of PDAs requires involvement from the clinical environments and an effective strategy for their systematic use [54]. The existence of high-quality patient information alone might not help if procedures for its use in the clinic are not in place and agreed upon. In the current study, existence and availability of a PDA suiting the tasks in the local environment was made an inclusion criterion. However, if these were not yet part of routine care, the nurses might have seen their implementation as part of decision coaching as too challenging.

Indeed, the lack of self-confidence in using decision aids does not fully explain the failure to adopt the behaviour, neither was it mentioned in the post-hoc inquiry assessing why Part B was not completed. The responses in the post-hoc inquiry revealed a collection of barriers. The startling contrast between the initial enthusiasm, positive attitudes and self-confidence and intentions to adopt decision coaching expressed in Part A and the actual behaviour later on needs to be better understood. The item evaluating the degree of self-confidence regarding PDA use showed that 50% only partly agreed and 31% disagreed. However, subjectively reported attitudes and intentions do not necessarily represent the active agents underlying subsequent behaviour.

Regarding training programme barriers, the primary barrier was participants' reluctance to audio record their consultations. This emerged as a major barrier to completing Part B of the training programme and measuring the coaching behaviour in order to give individualised feedback. To the extent that practical training and transferring knowledge to one’s own workplace is considered an essential part of the training, the barriers towards the sharing of insight into participants’ own decision coaching skills might in fact be seen as barriers to the implementation of SDM. The reluctance to share audio recordings warrants careful interpretation.

While it might be an indicator of the nurse's lack of readiness to adopt a new role as decision coach, the qualitative feedback from our post-hoc inquiry and previous research by Lenzen, Daniëls [52] suggests that practical and psychological barriers may be more significant factors. A basic requirement for decision coaching is establishing transparency and reducing power imbalances between healthcare providers and patients, such as the information monopoly on the side of the nurse and any kind of opacity [55]. However, as our study was not designed to investigate these particular attitudes, we do not know whether and to what extent this kind of barrier might have caused the dramatic dropout rate.

In the post-hoc interviews, aiming to elucidate the unexpected dropout rate, some nurses reported not pursuing the training because they were uncomfortable with audio recording their encounters; others reported lack of motivation as a key factor. These statements might also imply a lack of willingness to receive the following individualised feedback. Giving feedback on a video shared by the trainee is a well-known method and a BCT has the potential to impact positively upon building SDM skills [56, 57]. Golembiewski et al. claim that using recordings of consultations can “yield important insights about clinical practice without the inherent biases involved in asking patient or clinician participants to report the content, reflect on it, or rate the encounter retrospectively” [58]. Several studies report positive effects of audio/video feedback sessions on skill building [58–62]. Indeed, participants in an SDM training study by Ammentorp et al. indicated that the most beneficial aspect of the course was reviewing their own videos [60]. However, recording consultations can also be experienced as stressful. While the method is widely used in training physicians [56, 63], it seems less common in training nurses. A Norwegian study investigated making videos of consultations in the field of contraception counselling and found that the stress experienced by public health nursing students depended on the degree to which they felt well prepared professionally [64]. In a study conducted by Eeckhout et al. [59] evaluating the feasibility and acceptability of video recording during real-time patient encounters performed by general practitioner trainees, almost 70% of the trainees were positive about recording consultations and almost 90% noticed an improvement of their communication skills through observation and evaluation [59]. Nevertheless, as the nurses in our study, they felt uncomfortable and stressed due to the presence of the camera and assumed that patients felt the same way. This is supported by Dobber et al. who reported that the recording of conversations may be anxiety-inducing for the nurses [65]. Video feedback has also been used in SDM training for nurses. In a study by Rahn et al. [33], nurses stated that being videotaped during coaching sessions was burdensome and a study by Berger-Hoger et al. [19] was terminated partly due to the nurses not complying with the task of taking videos.

Using simulated patients could be an alternative to audio feedback [66], with the potential to overcome some of the reported barriers in our study, such as the lack of “suitable” patients or worrying about the patient’s anxiety. Previous studies evaluating the training for decision coaches’ behaviours used simulated patients with the audio recording conducted by the simulated patients [67]. However, this method is resource-consuming and does not provide a real-world SDM example. Other alternatives are to extend the training in order to practise audio recording; this may contribute to the delivery of an audio recording no longer representing an insurmountable exercise, or using self-assessment as a method of feedback [68]. Considering the positive evaluation of the classroom training and the subjectively perceived gain of skills, self-confidence and solid attitudes regarding patient involvement, an argument could also be made for omitting the audio recording. However, as discussed earlier, we would then lose an essential, well-documented training method [69].

Results from the present study indicate that the classroom training intervention was feasible and acceptable and the nurses’ reaction towards the training was good. Self-reported learning including attitudes, intentions and confidence regarding decision coaching was overall positively reported by the participants, which are the predecessors of actual behaviour [70]. Measuring Kirkpatrick’s level one, reaction and level two, learning is consistent with other pilot and feasibility studies, also within the field of SDM [49]. Evaluation results indicate a clear progression through different competency levels. At the knowledge level, 90% of participants understood SDM concepts and 84% grasped decision coaching principles. However, skill development showed variability given 74% felt confident in decision coaching and only 16% were fully confident using patient decision aids [71]. This suggests that while foundational knowledge is well-conveyed, advancing to complex skill application is more challenging. Participation was limited in Part B, highlighting difficulties in measuring/verifying higher competency levels during practical application.

In our study, we trained nurses from different units, which may have influenced our results. The majority of decision coaching studies are conducted with homogeneous groups [11], but our study stands out in this respect. Perhaps this is one of the challenges to success? Two reviews have previously stated that the interprofessional nature of SDM should be acknowledged for optimal implementation [7, 71], meaning that training whole departments, units, or teams may be more useful than training individuals from several medical domains. Results from our study also emphasises the importance of being more than one participant from one department. A realist review aimed to understand how and under what circumstances decision coaching works for people making healthcare decisions by Zhao et al. [16] reports that “Healthcare providers are likely to improve their knowledge and skills to implement decision coaching through training and practice”. This is supported by twenty-two papers that explicitly described the training for decision coaches. This underlines that others have succeeded with training healthcare professionals in decision coaching, some have even proven effective in increasing patient participation in SDM and informed choices [19]. Further research is needed to optimise training for nurses in decision coaching to reach our end goal of improving the quality of healthcare decisions.

## Conclusion

Decision coaching training was feasible to develop, guided by the KTA framework. Participants reported Part A as acceptable, relevant to their practice and recommended. The second part, including the delivery of an example of their decision coaching skills in consultations, was perceived as not feasible for various reasons. Further efforts are needed to resolve barriers related to applying or transferring skill training into clinical practice. Development of decision coaching skills requires, as a precondition, a culture of transparency and readiness for (behaviour) change. The preliminary evaluation offers valuable insights regarding the module’s feasibility and improvement needs, particularly with regard to the modes of delivery.

## Supplementary Information

Supplementary Material 1.

Supplementary Material 2.

Supplementary Material 3.

Supplementary Material 4.

## Data Availability

The datasets used and/or analysed during the current study are available from the corresponding author on reasonable request.

## Abbreviations

SDM: Shared decision making
OPDG: Ottawa Personal Decision Guide
PDA: Patient decision aids
BCTs: Behaviour change techniques
KTA: Knowledge-to-Action framework
MAPPIN’SDM: The Multifocal Approach to Sharing in Shared Decision Making

## References

[1] Pasient- og brukerrettighetsloven. Lov om pasient- og brukerrettigheter LOV-1999–07–02–631999.

[2] Helse- og omsorgsdepartementet. Meld. St. 7 - Nasjonal helse- og sykehusplan 2020–2023 2019.

[3] Helse Sør-Øst RHF. Regional utviklingsplan 2040 - Trender og satsingsområder. Oslo; 2022.

[4] Wennberg JE. Unwarranted variations in healthcare delivery: implications for academic medical centres. BMJ. 2002;325(7370):961–4. 12399352 10.1136/bmj.325.7370.961 PMC1124450

[5] Shay LA, Lafata JE. Where Is the Evidence? A Systematic Review of Shared Decision Making and Patient Outcomes. Med Decis Making. 2015;35(1):114–31. 25351843 10.1177/0272989X14551638 PMC4270851

[6] Stacey D, Legare F, Lewis K, Barry MJ, Bennett CL, Eden KB, et al. Decision aids for people facing health treatment or screening decisions. Cochrane Database Syst Rev. 2017;4:Cd001431. 10.1002/14651858.CD001431.pub5 PMC6478132 28402085

[7] Légaré F, Adekpedjou R, Stacey D, Turcotte S, Kryworuchko J, Graham ID, et al. Interventions for increasing the use of shared decision making by healthcare professionals. Cochrane Database of Systematic Reviews. 2018(7). 10.1002/14651858.CD006732.pub4 PMC6513543 30025154

[8] Diouf NT, Menear M, Robitaille H, Painchaud Guerard G, Legare F. Training health professionals in shared decision making: Update of an international environmental scan. Patient Educ Couns. 2016. 10.1016/j.pec.2016.06.008 27353259

[9] Legare F. Inventory of Shared Decision Making Programs for Healthcare Professionals: Université Laval; 2020 [Available from: http://www.decision.chaire.fmed.ulaval.ca/inventaire-formation-en.

[10] Siyam T, Shahid A, Perram M, Zuna I, Haque F, Archundia-Herrera MC, et al. A scoping review of interventions to promote the adoption of shared decision-making (SDM) among health care professionals in clinical practice. Patient Educ Couns. 2019;102(6):1057–66. 30642716 10.1016/j.pec.2019.01.001

[11] Jull J, Köpke S, Smith M, Carley M, Finderup J, Rahn AC, et al. Decision coaching for people making healthcare decisions. Cochrane Database Syst Rev. 2021;(11). 10.1002/14651858.CD013385.pub2 PMC8575556 34749427

[12] Direktoratet for ehelse. Samvalg Helsenorge.no2019 [updated 10.04.2024. Available from: https://helsenorge.no/samvalg.

[13] Bravo P, Härter M, McCaffery K, Giguère A, Hahlweg P, Elwyn G. Editorial: 20 years after the start of international Shared Decision-Making activities: Is it time to celebrate? Z Evid Fortbild Qual Gesundhwes. 2022;171:1–4. 10.1016/j.zefq.2022.05.009 35662496

[14] Kasper J, Lager AR, Rumpsfeld M, Kienlin S, Smestad KH, Brathen T, et al. Status report from Norway: Implementation of patient involvement in Norwegian health care. Z Evid Fortbild Qual Gesundhwes. 2017;123-124:75–80. 10.1016/j.zefq.2017.05.015 28546052

[15] Kasper J, Stensdal L-A, Kienlin S, Eiring Ø, Neset T, Andersen-Hollekim T, et al. New status report from Norway: Implementation of patient involvement in Norwegian health care. Zeitschrift fur Evidenz, Fortbildung und Qualitat im Gesundheitswesen. 2022. 10.1016/j.zefq.2022.04.021 35618624

[16] Zhao J, Jull J, Finderup J, Smith M, Kienlin SM, Rahn AC, et al. Understanding how and under what circumstances decision coaching works for people making healthcare decisions: a realist review. BMC Med Inform Decis Mak. 2022;22(1):265. 36209086 10.1186/s12911-022-02007-0 PMC9548102

[17] Stacey D, Murray MA, Légaré F, Sandy D, Menard P, O’ Connor A. Decision Coaching to Support Shared Decision Making: A Framework, Evidence, and Implications for Nursing Practice, Education, and Policy. Worldviews Evid Based Nurs. 2008;5(1):25–35. 18266768 10.1111/j.1741-6787.2007.00108.x

[18] Stacey D, Kryworuchko J, Bennett C, Murray MA, Mullan S, Legare F. Decision Coaching to Prepare Patients for Making Health Decisions: A Systematic Review of Decision Coaching in Trials of Patient Decision Aids. Med Decis Making. 2012;32(3):E22–33. 22505617 10.1177/0272989X12443311

[19] Berger-Hoger B, Liethmann K, Muhlhauser I, Haastert B, Steckelberg A. Nurse-led coaching of shared decision-making for women with ductal carcinoma in situ in breast care centers: A cluster randomized controlled trial. Int J Nurs Stud. 2019;93:141–52. 30925280 10.1016/j.ijnurstu.2019.01.013

[20] Waddell A, Lennox A, Spassova G, Bragge P. Barriers and facilitators to shared decision-making in hospitals from policy to practice: a systematic review. Implement Sci. 2021;16(1):74. 34332601 10.1186/s13012-021-01142-y PMC8325317

[21] Kienlin S, Amro A, Øverlie A, Kasper J. Opplæring i samvalg til sykepleiere i en master- og videreutdanning. Sykepleien Forskning. 2022;17.

[22] Kienlin S, Nytrøen K, Stacey D, Kasper J. Ready for shared decision making: Pretesting a training module for health professionals on sharing decisions with their patients. Journal of Evaluation in Clinical Practice. 2020;n/a(Special Issue Shared decision making). 10.1111/jep.13380 32114700

[23] Kienlin S, Stacey D, Nytrøen K, Grafe A, Kasper J. Ready for SDM- evaluation of an interprofessional training module in shared decision making - A cluster randomized trial. Patient Educ Couns. 2022;105(7):2307–14. 35365369 10.1016/j.pec.2022.03.013

[24] Kienlin S, Poitras M-E, Stacey D, Nytrøen K, Kasper J. Ready for SDM: evaluating a train-the-trainer program to facilitate implementation of SDM training in Norway. BMC Med Inform Decis Mak. 2021;21(1):140. 33931046 10.1186/s12911-021-01494-x PMC8086335

[25] Kienlin S. Klar for samvalg: Helse Sør-Øst RHF; 2021 [Available from: https://samvalg.no.

[26] Geiger F, Liethmann K, Reitz D, Galalae R, Kasper J. Efficacy of the doktormitSDM training module in supporting shared decision making - Results from a multicenter double-blind randomized controlled trial. Patient Educ Couns. 2017;100(12):2331–8. 28647064 10.1016/j.pec.2017.06.022

[27] Graham I, Logan J, Harrison MB, Straus SE, Tetroe J, Caswell W, et al. Lost in knowledge translation: Time for a map? J Contin Educ Heal Prof. 2006;26(1):13–24. 10.1002/chp.47 16557505

[28] Agbadjé TT, Riganti P, Adisso ÉL, Adekpedjou R, Boucher A, Nunciaroni AT, et al. Are shared decision making studies well enough described to be replicated? Secondary analysis of a Cochrane systematic review. PLoS ONE. 2022;17(3):e0265401. 35294494 10.1371/journal.pone.0265401 PMC8926249

[29] Michie S, Richardson M, Johnston M, Abraham C, Francis J, Hardeman W, et al. The behavior change technique taxonomy (v1) of 93 hierarchically clustered techniques: building an international consensus for the reporting of behavior change interventions. Ann Behav Med. 2013;46(1):81–95. 23512568 10.1007/s12160-013-9486-6

[30] von Elm E, Altman DG, Egger M, Pocock SJ, Gøtzsche PC, Vandenbroucke JP. The Strengthening the Reporting of Observational Studies in Epidemiology (STROBE) statement: guidelines for reporting observational studies. The Lancet. 2007;370(9596):1453–7. 10.1016/S0140-6736(07)61602-X 18064739

[31] Clark NM, Nelson, B. W., Valerio, M. A., Gong, Z. M., Taylor-Fishwick, J. C., & Fletcher, M. . Consideration of Shared Decision Making in Nursing: A Review of Clinicians’ Perceptions and Interventions. The Open Nursing Journal. 2009. 10.2174/1874434600903010065 PMC2765030 19855848

[32] University of Ottawa FoHS, School of Nursing. NSG 6133 – Decision Making in Clinical Practice: University of Ottawa,; 2022 [updated 08.09.2014. Available from: https://decisionaid.ohri.ca/uOttawa.html.

[33] Rahn AC, Kopke S, Backhus I, Kasper J, Anger K, Untiedt B, et al. Nurse-led immunotreatment DEcision Coaching In people with Multiple Sclerosis (DECIMS) - Feasibility testing, pilot randomised controlled trial and mixed methods process evaluation. Int J Nurs Stud. 2018;78:26–36. 28982479 10.1016/j.ijnurstu.2017.08.011

[34] Kienlin S, Kasper J, Liethmann K, Grafe A, Stacey D, Nytrøen K. Evaluation of an interprofessional training module in Shared Decision Making (Ready for SDM): a cluster randomized controlled trial. Patient Educ Couns. 2022. 10.1016/j.pec.2022.03.013 35365369

[35] Knowles M. Andragogikk: en kommende praksis for voksenopplæring. In: Berri S, editor. I Illeris K. Tekster om voksenlæring. Danmark: Roskilde Univeritetsforlag; 2005. p. 59–72.

[36] Forskrift om nasjonal retningslinje for sykepleierutdanning, (2019).

[37] Bloom BS, Engelhart MD, Furst EJ, Hill WH, Krathwohl DR. Taxonomy of educational objectives: The classification of educational goals. Handbook 1: Cognitive domain: Longman New York; 1956.

[38] Stacey D, O’Connor AM, Graham ID, Pomey MP. Randomized controlled trial of the effectiveness of an intervention to implement evidence-based patient decision support in a nursing call centre. J Telemed Telecare. 2006;12(8):410–5. 17227607 10.1258/135763306779378663

[39] Kienlin S, Stacey D, Kasper J. Ready for SDM – development of a modularized meta- curriculum for training healthcare professionals in shared decision-making. BMJ Evidence-Based Medicine. 2024;29(Suppl 1):A59.

[40] Michie S, Johnston M. Theories and techniques of behaviour change: Developing a cumulative science of behaviour change. Health Psychol Rev. 2012;6(1):1–6.

[41] Hoffmann TC, Glasziou PP, Boutron I, Milne R, Perera R, Moher D, et al. Better reporting of interventions: template for intervention description and replication (TIDieR) checklist and guide. BMJ : British Medical Journal. 2014;348:g1687. 24609605 10.1136/bmj.g1687

[42] Kasper J, Hoffmann F, Heesen C, Köpke S, Geiger F. MAPPIN’SDM – The Multifocal Approach to Sharing in Shared Decision Making (MAPPIN’SDM). PLoS ONE. 2012;7(4):e34849. 22514677 10.1371/journal.pone.0034849 PMC3325952

[43] Stacey D, Légaré F, Boland L, Lewis KB, Loiselle MC, Hoefel L, et al. 20th Anniversary Ottawa Decision Support Framework: Part 3 Overview of Systematic Reviews and Updated Framework. Med Deci Making. 2020;40(3):379–98. 10.1177/0272989X20911870 32428429

[44] O’Connor, Stacey D, Jacobsen. Ottawa Personal Decision Guides Canada.: Ottawa Hospital Research Institute & University of Ottawa; 2015 [updated 03.01.2020. Available from: https://decisionaid.ohri.ca/decguide.html.

[45] Kienlin S, Kristiansen M, Ofstad E, Liethmann K, Geiger F, Joranger P, et al. Validation of the Norwegian version of MAPPIN’SDM, an observation-based instrument to measure shared decision-making in clinical encounters. Patient Educ Couns. 2017;100(3):534–41. 28029570 10.1016/j.pec.2016.10.023

[46] Forner D, Noel CW, Boland L, Pieterse AH, Borkhoff CM, Hong P. The Multifocal Approach to Sharing in Shared Decision Making: A Critical Appraisal of the MAPPIN’SDM. Med Decis Making. 2021;42(1):114–24. 33966516 10.1177/0272989X211010738

[47] Kirkpatrick JD, Kirkpatrick WK. Kirkpatrick’s four levels of training evaluation. Alexandria, United States: ATD Press; 2016. p. 256.

[48] Hsieh H-F, Shannon SE. Three Approaches to Qualitative Content Analysis. Qual Health Res. 2005;15(9):1277–88. 16204405 10.1177/1049732305276687

[49] Müller E, Strukava A, Scholl I, Härter M, Diouf NT, Légaré F, et al. Strategies to evaluate healthcare provider trainings in shared decision-making (SDM): a systematic review of evaluation studies. BMJ Open. 2019; 9(6):e026488. Available from: http://bmjopen.bmj.com/content/9/6/e026488.abstract. 10.1136/bmjopen-2018-026488 PMC6596948 31230005

[50] Joseph-Williams N, Elwyn G, Edwards A. Knowledge is not power for patients: a systematic review and thematic synthesis of patient-reported barriers and facilitators to shared decision making. Patient Educ Couns. 2014;94(3):291–309. 24305642 10.1016/j.pec.2013.10.031

[51] Scholl I, LaRussa A, Hahlweg P, Kobrin S, Elwyn G. Organizational- and system-level characteristics that influence implementation of shared decision-making and strategies to address them - a scoping review. Implement Sci. 2018;13(1):40. 29523167 10.1186/s13012-018-0731-z PMC5845212

[52] Lenzen SA, Daniëls R, van Bokhoven MA, van der Weijden T, Beurskens A. What makes it so difficult for nurses to coach patients in shared decision making? A process evaluation. Int J Nurs Stud. 2018;80:1–11. 29331655 10.1016/j.ijnurstu.2017.12.005

[53] Oppdragsdokument for 2017, (2017).

[54] Kasper J, Rumpsfeld M, Lauritzen M, Johnsen AG, Måseide AK, Lager AR, et al. The Decision Aid Factory (DAfactory) – between prototype and series production. The 9th International Shared Decision Making conference; Lyon, France2017.

[55] Fossa AJ, Bell SK, DesRoches C. OpenNotes and shared decision making: a growing practice in clinical transparency and how it can support patient-centered care. J Am Med Inform Assoc. 2018;25(9):1153–9. 29982659 10.1093/jamia/ocy083 PMC7646890

[56] Kasper J, Liethmann K, Heesen C, Reissmann DR, Geiger F. Training doctors briefly and in situ to involve their patients in making medical decisions-Preliminary testing of a newly developed module. Health Expect. 2017;20(6):1254–63. 28521082 10.1111/hex.12565 PMC5689231

[57] Agbadjé TT, Elidor H, Perin MS, Adekpedjou R, Légaré F. Towards a taxonomy of behavior change techniques for promoting shared decision making. Implement Sci. 2020;15(1):67. 32819410 10.1186/s13012-020-01015-w PMC7439658

[58] Golembiewski EH, Espinoza Suarez NR, Maraboto Escarria AP, Yang AX, Kunneman M, Hassett LC, et al. Video-based observation research: A systematic review of studies in outpatient health care settings. Patient Education and Counseling. 2022. 10.1016/j.pec.2022.09.017 36207219

[59] Eeckhout T, Gerits M, Bouquillon D, Schoenmakers B. Video training with peer feedback in real-time consultation: acceptability and feasibility in a general-practice setting. Postgrad Med J. 2016;92(1090):431–5. 26842970 10.1136/postgradmedj-2015-133633 PMC4975814

[60] Ammentorp J, Wolderslund M, Timmermann C, Larsen H, Steffensen KD, Nielsen A, et al. How participatory action research changed our view of the challenges of shared decision-making training. Patient Educ Couns. 2018;101(4):639–46. 29137836 10.1016/j.pec.2017.11.002

[61] Pehrson C, Banerjee SC, Manna R, Shen MJ, Hammonds S, Coyle N, et al. Responding empathically to patients: Development, implementation, and evaluation of a communication skills training module for oncology nurses. Patient Educ Couns. 2016;99(4):610–6. 26686992 10.1016/j.pec.2015.11.021 PMC4962546

[62] Caris-Verhallen WMCM, Kerkstra A, Bensing JM, Grypdonck MHF. Effects of video interaction analysis training on nurse–patient communication in the care of the elderly. Patient Educ Couns. 2000;39(1):91–103. 11013551 10.1016/s0738-3991(99)00094-4

[63] Nilsen S, Baerheim A. Feedback on video recorded consultations in medical teaching: why students loathe and love it - a focus-group based qualitative study. BMC medical education. 2005;5:28-. 10.1186/1472-6920-5-28 PMC1190180 16029509

[64] Olufsen V, Aune I. Videopptak som læringsaktivitet i praktiske studier kan både fremme og hemme læring. Sykepleien Forskning. 2015;10:72–80.

[65] Dobber J, Latour C, Snaterse M, van Meijel B, Ter Riet G, Scholte Op Reimer W, et al. Developing nurses’ skills in motivational interviewing to promote a healthy lifestyle in patients with coronary artery disease. Eur J Cardiovasc Nursing. 2019;18(1):28–37. 10.1177/1474515118784102 29905499

[66] Müller E, Diesing A, Rosahl A, Scholl I, Härter M, Buchholz A. Evaluation of a shared decision-making communication skills training for physicians treating patients with asthma: a mixed methods study using simulated patients. BMC Health Serv Res. 2019;19(1):612. 31470856 10.1186/s12913-019-4445-y PMC6716840

[67] Stacey D, Pomey M-P, O’Connor AM, Graham ID. Adoption and sustainability of decision support for patients facing health decisions: an implementation case study in nursing. Implement Sci. 2006;1(1):17. 16930476 10.1186/1748-5908-1-17 PMC1564029

[68] National institute for Health and Care Exellence. Shared decision making - NICE guideline [NG197]. NICE National institute for Health and Care Exellence; 2021 17 June 2021.

[69] Hammoud MM, Morgan HK, Edwards ME, Lyon JA, White C. Is video review of patient encounters an effective tool for medical student learning? A review of the literature. Adv Med Educ Pract. 2012;3:19–30. 23761999 10.2147/AMEP.S20219 PMC3650868

[70] Légaré F, Freitas A, Thompson-Leduc P, Borduas F, Luconi F, Boucher A, et al. The majority of accredited continuing professional development activities do not target clinical behavior change. Acad Med. 2015;90(2):197–202. 25354076 10.1097/ACM.0000000000000543

[71] Joseph-Williams N, Abhyankar P, Boland L, Bravo P, Brenner AT, Brodney S, et al. What Works in Implementing Patient Decision Aids in Routine Clinical Settings? A Rapid Realist Review and Update from the International Patient Decision Aid Standards Collaboration. Medical Decision Making. 2021;0(0):0272989X20978208. 10.1177/0272989X20978208 PMC8474331 33319621

