# Supplementary material for: Development and preliminary evaluation of a decision coach training module for nurses in Norway
Source: BMC Nurs. 2025 Feb 10;24:152. doi: 10.1186/s12912-024-02569-6 (PMC11808981; doi:10.1186/s12912-024-02569-6)
Supplement: Supplementary file 2 — Supplementary Material 2. [file 12912_2024_2569_MOESM2_ESM.docx]

**
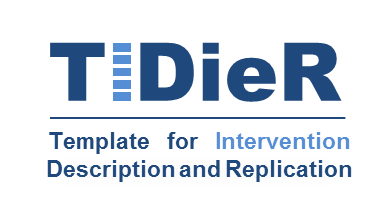
 The TIDieR (Template for Intervention Description and Replication) Checklist*:**

Information to include when describing an intervention and the location of the information

| 1. **BRIEF NAME**: | **Where located** |
| --- | --- |
| 1. INTERVENTION: Ready for SDM – Decision coach training module nurses. | Page 1 |
| 1. **WHY** |  |
| The main learning objective of the “Ready for SDM: decision coaching” training module was to build the knowledge and skills they need to help patients prepare to actively participate in making health decisions. The module also aimed to enable nurses to use existing decision aids relevant to their local medical domains.  The development of the meta-curriculum (Ready for SDM) was done in accord with the Knowledge-to-Action framework (KTA), based on MAPPIN´SDM (Multifocal Approach to the Sharing in SDM) as its underpinning concept of SDM quality. Single components regarding the nurses role in interprofessional SDM were developed or adjusted inspired by the Ottawa Decision Support Framework (ODSF) and the Framework for Decision Coach-Mediated Shared Decision Making. | Page 5-6  Page 6 and table 1. |
| 1. **WHAT** |  |
| **MATERIALS:** Learning materials from the meta-curriculum was used/adjusted for this intervention (see table 1).  **EDUCATIONAL STRATEGIES:** To achieve the learning goals of the module (see Table 1) and to achieve consolidation, the developers decided to have a breadth of learning activities such as lectures, group work, think-pair-share (TPS), reflection exercises and group/individual feedback.  Additional it was decided to adhere to evidence-based behavior change techniques (BCTs).  **INCENTIVES:** Participants were not paid and there were otherwise no financial incentives. | Page 7-11 and . Table 1, 2 & 3 |
| 1. **WHO PROVIDED** |  |
| **INSTRUCTORS:** All training was delivered by SK, a registered nurse with a master’s degree in Health and Empowerment and a Ph.D student. She was trained at in the ODSF at the University of Ottawa in a graduate course and is a certified MAPPIN´SDM observer. | Page 12 |
| 1. **HOW** |  |
| DELIVERY: Given the structures and conditions in Norwegian hospitals, we planned a one-day module with a practical exercise and virtual or face-to-face follow-up. | Page 7. |
| 1. **WHERE** |  |
| ENVIRONMENT: The intervention was conducted at two study sites (hospitals) within the Northern and the South-Eastern Norway Regional Health Authorities. The training program was designed for a maximum of ten nurses per course and counted as working time. | Page 14. |
| **WHEN and HOW MUCH** |  |
| Part A of the intervention occurred in a hospital meeting room at the University Hospital of North Norway (UNN) in Tromsø (Northern Regional Health Authority) in December 2019, and at the Akershus University Hospital (South-Eastern Norway Regional Health Authority) in January 2020.  SCHEDULE:  **Part A: Decision coaching skills (6 hours)**  Part A focuses on SDM basics, learning to use a decision aid and role-play.  See table 1.  **Part B: Decision coaching practice and personal evaluation (1 hour)**  The goal of Part B was that nurses 1) apply the knowledge and skills from Part A by providing decision coaching to an actual patient with decisional needs in their own clinical practice while audiotaping the encounter, 2) analyze the decision coaching they provided, and 3) receive individualized feedback on their audiotaped decision coaching. | Table 1  Page 7-11  Page 14. |
| **TAILORING** |  |
| The intervention was tailored to the target group based on previous piloting, evaluation and educational strategies. | Page 7-11 |
| **MODIFICATIONS** |  |
| Modifications were made to the intervention after the piloting of the training components.  There were no unplanned changes during the course. | Page 6 |
| **HOW WELL** |  |
| ATTENDANCE:  All participants completed part A of the training, however only one participant completed part B. | Page 14-15 |

Hoffmann TC, Glasziou PP, Boutron I, et al. Better reporting of interventions: template for intervention description and replication (TIDieR) checklist and guide. BMJ 2014; 348: g1687.
