# Supplementary material for: Development and preliminary evaluation of a decision coach training module for nurses in Norway
Source: BMC Nurs. 2025 Feb 10;24:152. doi: 10.1186/s12912-024-02569-6 (PMC11808981; doi:10.1186/s12912-024-02569-6)
Supplement: Supplementary file 3 — Supplementary Material 3. [file 12912_2024_2569_MOESM3_ESM.docx]

MAPPIN'SDM self-observation: healthcare professional

| This self-observation sheet should be used after a consultation in which a medical decision was discussed or made. The decision may concern diagnostics, treatment or follow-up (e.g. choosing between different medications, between medicine or surgery, diagnostic examinations or choosing between treatment or no treatment.The purpose is for you to assess for yourself the extent to which the patient was involved in the decision. **Follow the instructions below:** |
| --- |
| State the problem to which the decision applied, e.g. the choice between different medications for the treatment of depression, or treatment x and operation y.**Write here:** …………………………………………………………………………………………………………………………………... |
| **Review all the shared decision-making criteria on page 2** and tick the answer option that best suits your experience of the conversation. |
| **After answering the questions on page 2, try answering the following**:  Could you improve some of the steps so that the patient could be involved to a greater extent? If so, what specifically could you have done differently?  **Write here:**  ………………………………………………………………………………………………………………………………...... |
|  |

|  |
| --- |

The quality criteria used in the decision-making process, which is often only part of a conversation.

|  | **Quality criteria** | **Self-assessment** | | | | |
| --- | --- | --- | --- | --- | --- | --- |
| **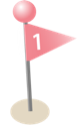**  **Defining the problem** | **The patient and I** agreed on a specific issue that requires a decision. | **Totally disagree** | **Slightly disagree** | **Neither nor** | **Slightly agree** | **Totally agree** |

| **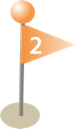**  **Key- messages** | **The patient and I** noted that there is more than one way to deal with the specific problem and that it is therefore up to the patient to decide which option is best. | **Totally disagree** | **Slightly disagree** | **Neither nor** | **Slightly agree** | **Totally agree** |
| --- | --- | --- | --- | --- | --- | --- |

| **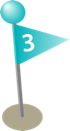**  **Options (structure)** | **The patient and I** have listed and reviewed the options in a structured way that is easy to understand and easy to remember (if relevant, also *do nothing*) | **Totally disagree** | **Slightly disagree** | **Neither nor** | **Slightly agree** | **Totally agree** |
| --- | --- | --- | --- | --- | --- | --- |

| **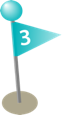**  **Options (content)** | **The patient and I** clarified the potential benefits and harms of each option (if relevant, of *doing nothing*). | **Totally disagree** | **Slightly disagree** | **Neither nor** | **Slightly agree** | **Totally agree** |
| --- | --- | --- | --- | --- | --- | --- |

| **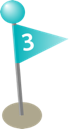**  **Options**  **(information quality)** | **The patient and I** have specified the likelihood of benefits and harms to occur in an understandable way (using numbers indication absolute risk or risk reduction) and referred to sources and the quality of the sources. | **Totally disagree** | **Slightly disagree** | **Neither nor** | **Slightly agree** | **Totally agree** |
| --- | --- | --- | --- | --- | --- | --- |

| **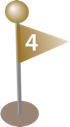**  **Deliberation** | **The patient and I** identified the patient's values, preferences, expectations and concerns and weighed the pros and cons of the options against each other. | **Totally disagree** | **Slightly disagree** | **Neither nor** | **Slightly agree** | **Totally agree** |
| --- | --- | --- | --- | --- | --- | --- |

| **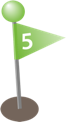**  **Decision** | **The patient and I** made it clear that they are now in the process of deciding among the available options (if applicable, postpone the decision). | **Totally disagree** | **Slightly disagree** | **Neither nor** | **Slightly agree** | **Totally agree** |
| --- | --- | --- | --- | --- | --- | --- |

| **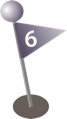**  **Arrangements** | **The patient and I** discussed what to do next (e.g., measures to implement the decision, timing of check-ups, how to evaluate the decision). | **Totally disagree** | **Slightly disagree** | **Neither nor** | **Slightly agree** | **Totally agree** |
| --- | --- | --- | --- | --- | --- | --- |

**Additional criteria for the decision-making process until the decision has been made**

| **Approach to communication** | **The patient and I** clarified any need to adjust the mode of communication including information giving, e.g. by using diagrams, images, film, interpreter etc. | **Totally disagree** | **Slightly disagree** | **Neither nor** | **Slightly agree** | **Totally agree** |
| --- | --- | --- | --- | --- | --- | --- |

| **Evaluation of patient understanding** | **The patient and I** clarified whether the patient understood the information I was conveying. | **Totally disagree** | **Slightly disagree** | **Neither nor** | **Slightly agree** | **Totally agree** |
| --- | --- | --- | --- | --- | --- | --- |

| **Evaluation of healthcare personnel's understanding** | **The patient and I** clarified whether I understood the patient's point of view. | **Totally disagree** | **Slightly disagree** | **Neither nor** | **Slightly agree** | **Totally agree** |
| --- | --- | --- | --- | --- | --- | --- |

1. Kasper J, Hoffmann F, Heesen C, Köpke S, Geiger F. MAPPIN'SDM – The Multifocal Approach to Sharing in Shared Decision Making (MAPPIN'SDM). PLoS ONE (2012).
2. Kienlin S, Kristiansen M, Ofstad E, Liethmann K, Geiger F, Joranger P, et al. Validation of the Norwegian version of MAPPIN'SDM, an observation-based instrument to measure shared decision-making in clinical encounters. Patient Educ Couns (2017).
