# Supplementary material for: Development and preliminary evaluation of a decision coach training module for nurses in Norway
Source: BMC Nurs. 2025 Feb 10;24:152. doi: 10.1186/s12912-024-02569-6 (PMC11808981; doi:10.1186/s12912-024-02569-6)
Supplement: Supplementary file 4 — Supplementary Material 4. [file 12912_2024_2569_MOESM4_ESM.docx]

## **Online questionnaire**

## **Post-classroom training:**

Thank you for taking the time to evaluate the decision coaching module. Your responses will help us refine the training to better meet the learning needs of people like you.

1. Sex
   - Female
   - Male
2. Age
   - <30
   - 30-50
   - >50+
   - Do not wish to state
3. What is your profession?
   - Registered nurse
   - Physiotherapist
   - Other, please specify:
4. Which field/department do you work in? ___________________
5. Years of clinical practice
   - < 1 year
   - 1-5 years
   - 6-15 years
   - 15+years
6. I consider what I learned during the course to be relevant for carrying out my work in the department

Strongly disagree 1 2 3 4 Strongly agree

1. Think back on the course, what has been most useful for you?_______
2. I would recommend this course to other nurses who have PDAs available at their department

yes no unsure

### What are your suggestions for improving the training___________________________________________________________

1. I perceived the concept of SDM as understandable

Strongly disagree 1 2 3 4 Strongly agree

1. I perceived the concept of decision coaching as understandable

Strongly disagree 1 2 3 4 Strongly agree

### You think it is desirable to involve patients in health decisions

Strongly disagree 1 2 3 4 Strongly agree

### My current competence in decision coaching is good

Strongly disagree 1 2 3 4 Strongly agree

1. I consider it likely that I will carry out coaching with my patients during the next 6 months, in addition to the home-task of conducting a coaching session

Strongly disagree 1 2 3 4 Strongly agree

### I feel able to provide decision coaching while using a decision aid

Strongly disagree 1 2 3 4 strongly agree

1. What additional support do you need to make decision coaching a routine in your department?____________________________________________
2. What do you think stands in the way of you to provide decision coaching?______________________________________________________
3. How likely is it that you will have the opportunity to carry out decision coaching in your department?

Very likely, fairly likely, not sure, unlikely, very unlikely.
